# Putative causal relations among gut flora, serums metabolites and arrhythmia: a Mendelian randomization study

**DOI:** 10.1186/s12872-023-03703-z

**Published:** 2024-01-11

**Authors:** Kaiyuan Li, Peng Liu, Miao Liu, Jun Ye, Li Zhu

**Affiliations:** 1 Graduate School of Dalian Medical University, Dalian Medical University, Dalian, China; 2 Department of Cardiovascular Medicine, The Second Affiliated Hospital of Nanchang University, Nanchang, China; 3 Department of Cardiovascular Medicine, Central Hospital Affiliated to Shandong First Medical University, Jinan, China; 4 Department of Cardiovascular Medicine, The Affiliated Taizhou People’s Hospital of Nanjing Medical University, No. 399 Hailing South Road, Taizhou, Jiangsu Province China

**Keywords:** Gut flora, Serums metabolites, Arrhythmia, GWAS, Mendelian randomization

## Abstract

**Background:**

The pathogenesis of cardiac arrhythmias is multifaceted, encompassing genetic, environmental, hemodynamic, and various causative factors. Emerging evidence underscores a plausible connection between gut flora, serum metabolites, and specific types of arrhythmias. Recognizing the role of host genetics in shaping the microbiota, we employed two-sample Mendelian randomization analyses to investigate potential causal associations between gut flora, serum metabolites, and distinct arrhythmias.

**Methods:**

Mendelian randomization methods were deployed to ascertain causal relationships between 211 gut flora, 575 serum metabolites, and various types of arrhythmias. To ensure the reliability of the findings, five complementary Mendelian randomization methods, including inverse variance weighting methods, were employed. The robustness of the results was scrutinized through a battery of sensitivity analyses, incorporating the Cochran Q test, leave-one-out test, and MR-Egger intercept analysis.

**Results:**

Eighteen gut flora and twenty-six serum metabolites demonstrated associations with the risk of developing atrial fibrillation. Moreover, ten gut flora and fifty-two serum metabolites were linked to the risk of developing supraventricular tachycardia, while eight gut flora and twenty-five serum metabolites were associated with the risk of developing tachycardia. Additionally, six gut flora and twenty-one serum metabolites exhibited associations with the risk of developing bradycardia.

**Conclusion:**

This study revealed the potential causal relationship that may exist between gut flora, serum metabolites and different cardiac arrhythmias and highlights the need for further exploration. This study provides new perspectives to enhance diagnostic and therapeutic strategies in the field of cardiac arrhythmias.

**Supplementary Information:**

The online version contains supplementary material available at 10.1186/s12872-023-03703-z.

## Introduction

Arrhythmia is a disorder of the origin and/or conduction of cardiac activity resulting in an abnormal rate and/or rhythm of the heartbeat, which may result in sudden death from a sudden onset, or may continue to involve the heart and cause it to fail [1–3]. Arrhythmias can be categorized into bradyarrhythmias and tachyarrhythmias according to the heart rate, with bradyarrhythmias mainly characterized by bradycardia and atrioventricular block, and tachyarrhythmias mainly characterized by tachycardia, supraventricular tachycardia, ventricular fibrillation, and atrial fibrillation [4]. Atrial fibrillation causes hemodynamic abnormalities and increases the morbidity and mortality of thromboembolic events, while ventricular arrhythmias can lead to palpitations or blackouts and even sudden cardiac death, as all types of arrhythmias add to the global economic burden [5–7].

Gut flora is a group of microorganisms that are planted in the human intestinal tract and are interdependent with the human body over a long period of time, which is very large in number and is known as the "second genome" of the human body [8, 9]. Gut flora is not only involved in the digestion and absorption of food, but also in the regulation of the body's immunity and the maintenance of the body's health. Gut flora can secrete a variety of bioactive metabolites including short-chain fatty acids, lipopolysaccharides, trimethylamine oxide and bile acids to regulate the corresponding target organs [10, 11]. The lack or disorder of gut flora seriously affects the body's decomposition of polysaccharides in food and absorption of lipids, leading to dysfunction of the liver and adipose tissue, and inducing a series of metabolic diseases, such as obesity, fatty liver, type 2 diabetes, and cardio-cerebral vascular disease [12]. Recently, several studies have proposed a new concept of the cardio-intestinal axis, suggesting a role for the gut microbiota in the development of various cardiovascular diseases such as coronary artery disease, hypertension and heart failure [13]. It has been noted that a wide range of bacteria have been identified in atherosclerotic plaques, and reduced diversity of the gut microbiota has been observed in patients with heart failure, but studies on gut microbial profiles and various types of arrhythmias are unclear [14].

Serum metabolites originate from various metabolic pathways in the body. The gut flora produces a wide variety of metabolites, which have a systemic effect on the body and can be used to assess the physiological and pathological state of the body, and some of the serum metabolites can also be used as biomarkers to assist in the diagnosis of some clinical diseases [15–17].

Genome-wide association studies (GWAS) validate phenotype-genotype associations by detecting genetic variation in large sample populations [18]. Mendelian laws describe the random distribution of genetic variants during meiosis, and in the absence of randomized clinical trials (RCTs), Mendelian randomization(MR) studies are natural RCTs [19]. We screened instrumental variables (IVs) for genetic variation through GWAS to explore potential causal relationships between exposure factors and disease.

The aim of this study was to investigate the potential causal relationship between gut microbiota and metabolites and various cardiac arrhythmias through a two-sample MR.

## Materials and methods

### Study design overview

**Fig. 1:**
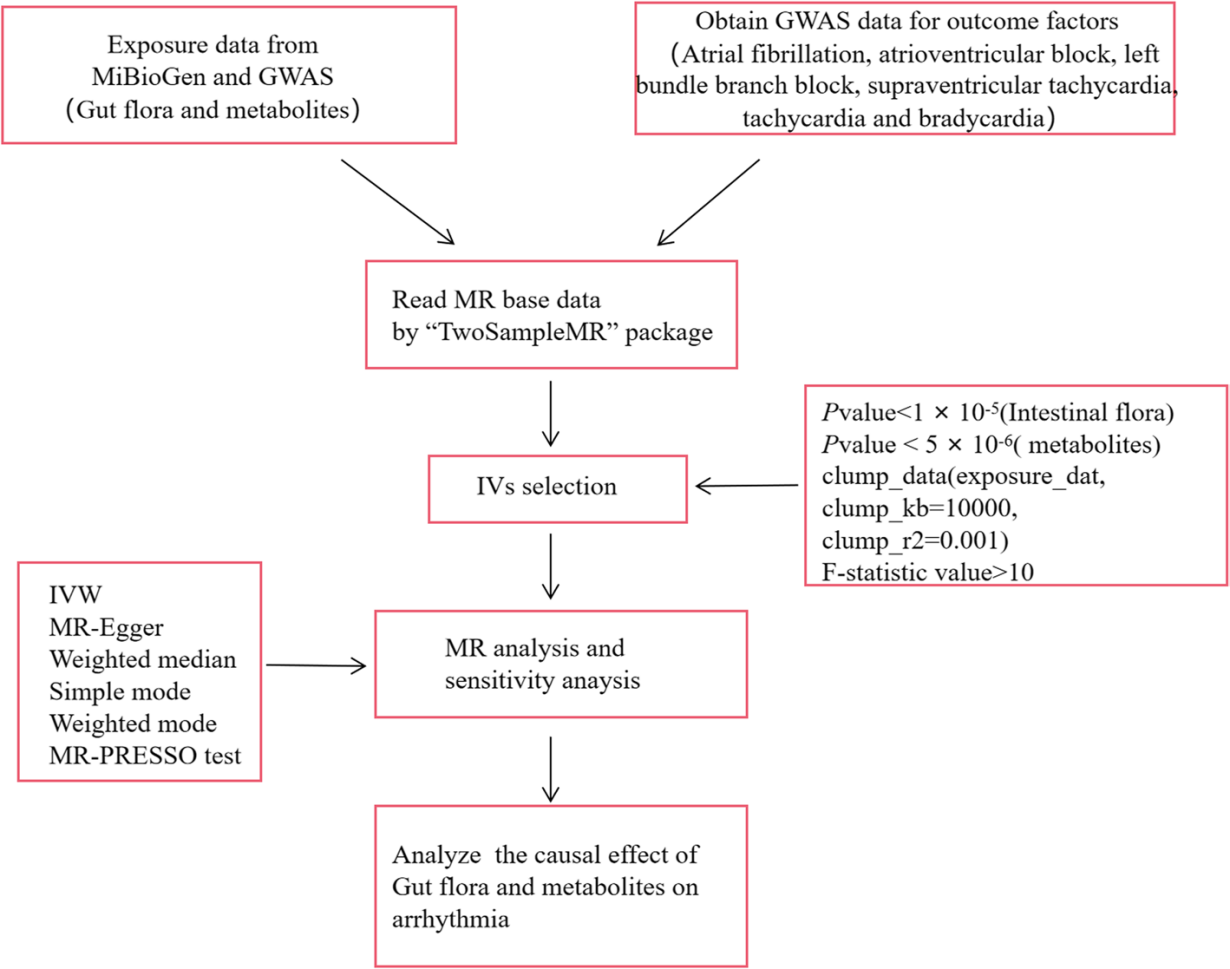
Overall flow chart of this study

In this study, a two-sample MR was used to investigate the causal associations of gut microbiota and serum metabolites with the risk of several types of arrhythmic diseases. Meanwhile, the MR design followed three assumptions. First, there must be a strong association between SNPs and exposure; second, SNPs should not be associated with outcomes through confounders; and third, SNPs should not directly influence outcomes. The total dataset included in this study was ethically approved by the relevant ethical review boards, and all patients involved were aware of and signed an informed consent form.(Fig. 1) [20].

### Date sources

#### Exposure sources and Genetic instrument selection

Gut flora data were downloaded from the MiBioGen (https://mibiogen.gcc.rug.nl/) website. A total of 18,340 16S rRNA gene sequencing profiles and genotyping data were collected from 18,340 subjects in 11 countries, including Asia and Europe, to identify genetic loci that influence the relative abundance or presence of microbial taxa. A total of 211 traits in 35 families, 20 orders, 16 phyla, 9 orders and 131 genera were included in the gut flora data. Considering that few SNPs loci with *P* < 1 × 10^–8^ were available for gut flora, SNPs loci with *P* < 1 × 10^–5^ were selected, and the loci obtained from the screening were used as instrumental variables in place of the clinical risk exposure factor gut flora (Supplementary Table S1) [10, 21]. Data from SNPs with chained unbalanced aggregates were subsequently removed, with removal conditioned on LD (*r*^2^ < 0.001, distance = 10,000 kb). SNPs that did not belong to a specific bacterial trait were excluded. Serum metabolite data were downloaded from the GWAS data, and a total of 575 metabolite-associated exposures were collected, and SNPs were screened based on *P* < 1 × 10^–5^, *r*^2^ < 0.001, distance < 10 000 kb (Supplementary Table S2) [22].

#### Outcome sources

Data on atrial fibrillation were derived from a GWAS of susceptibility genes published in 2018 (PMID: 30,061,737), the study included 60,620 patients with atrial fibrillation, 970,216 healthy controls, and contained 33,519,037 SNPs [23]. Data for the remaining types of arrhythmic disease were obtained from studies in the UK Biobank and Finnish databases, with studies of supraventricular tachycardia comprising 1,306 patients, 461,704 healthy controls, and 9,851,867 SNPs. studies of bradycardia comprised 1,005 patients with supraventricular tachycardia, 462,005 healthy controls, and 9,851,867 SNPs. studies of bradycardia comprised 1,254 patients, 461,756 healthy controls, and 9,851,867 SNPs. The study of bradycardia contained 1,254 patients, 461,756 healthy controls, and 9,851,867 SNPs. The study of atrioventricular block contained 5,536 patients, 286,109 healthy controls, and 16,380,173 SNPs. The study of left bundle branch block consisted of 1,918 patients, 286,109 healthy controls, and 16,380,167 SNPs. The study of right bundle branch block consisted of 9,545 patients, 286,109 healthy controls, and 16,380,175 SNPs. The study of right bundle branch block consisted of 9,545 patients, 286,109 healthy controls, and 16,380,175 SNPs (Table 1). In this study, the diagnostic criteria for all types of arrhythmias adhere to the International Classification of Diseases, Tenth Revision (ICD-10).

**Table 1:** Source of GWAS data for all types of arrhythmias

Exposure/Outcome	Year	Author	Participants	Number of SNPs	Web Source if Publicly
Atrial fibrillation	2018	Nielsen JB (PMID: 30,061,737)	1,030,836 individuals (60,620 use cases and 970,216 controls) of European ancestry	33,519,037	https://gwas.mrcieu.ac.uk/datasets/ebi-a-GCST006414/ (Access time:July 22, 2023)
Supraventricular tachycardia	2018	Ben Elsworth (PMID: 34,662,886)	463,010 individuals (1,306 use cases and 461,704 controls) of European ancestry	9,851,867	https://gwas.mrcieu.ac.uk/datasets/ukb-b-11748/ (Access time:July 22, 2023)
Tachycardia	2018	Ben Elsworth (PMID: 34,737,426)	463,010 individuals (1,005 cases and 462,005 controls) of European ancestry	9,851,867	https://gwas.mrcieu.ac.uk/datasets/ukb-b-17309/ (Access time:July 22, 2023)
Bradycardia	2018	Ben Elsworth (PMID: 34,737,426)	463,010 individuals (1,254 cases and 461,756 controls) of European ancestry	9,851,867	https://gwas.mrcieu.ac.uk/datasets/ukb-b-11664/ (Access time:July 22, 2023)
Atrioventricular block	2021	NA	159,099 individuals (2,388 cases and 156,711 controls) of European ancestry	16,380,173	https://risteys.finregistry.fi/endpoints/I9_AVBLOCK(Access time:July 22, 2023)
left bundle branch block	2021	NA	289,683 individuals (3,574 cases and 286,109 controls) of European ancestry	16,380,167	https://risteys.finregistry.fi/endpoints/I9_LBBB (Access time:July 22, 2023)

Source of GWAS data for all types of arrhythmias

### Statistical methods

In this study, the association between gut flora and various types of arrhythmias was analyzed by inverse variance weighting (IVW). The mean IVW values of SNP ratio estimates were derived by regressing SNP—gut flora on SNP—arrhythmia associations. The weighted median method (WME), MR-Egger regression test, Simple mode, and Weighted mode were used as supplements. Cochrane's Q was used to test the snp-related heterogeneity of each bacterial trait. In addition, sensitivity analyses were performed using the MR-Egger intercept test and leave-one-out analysis. *p*-values from the MR-Egger intercept test were used as an indicator of horizontal pleiotropy (*p* < 0.05 statistically significant). Leave-one-out analysis was used to identify potential pleiotropic effects from individual SNPs.

## Results

### Causal relationship between gut flora and various types of cardiac arrhythmias

#### Causal relationship between gut flora and atrial fibrillation

**Fig. 2:**
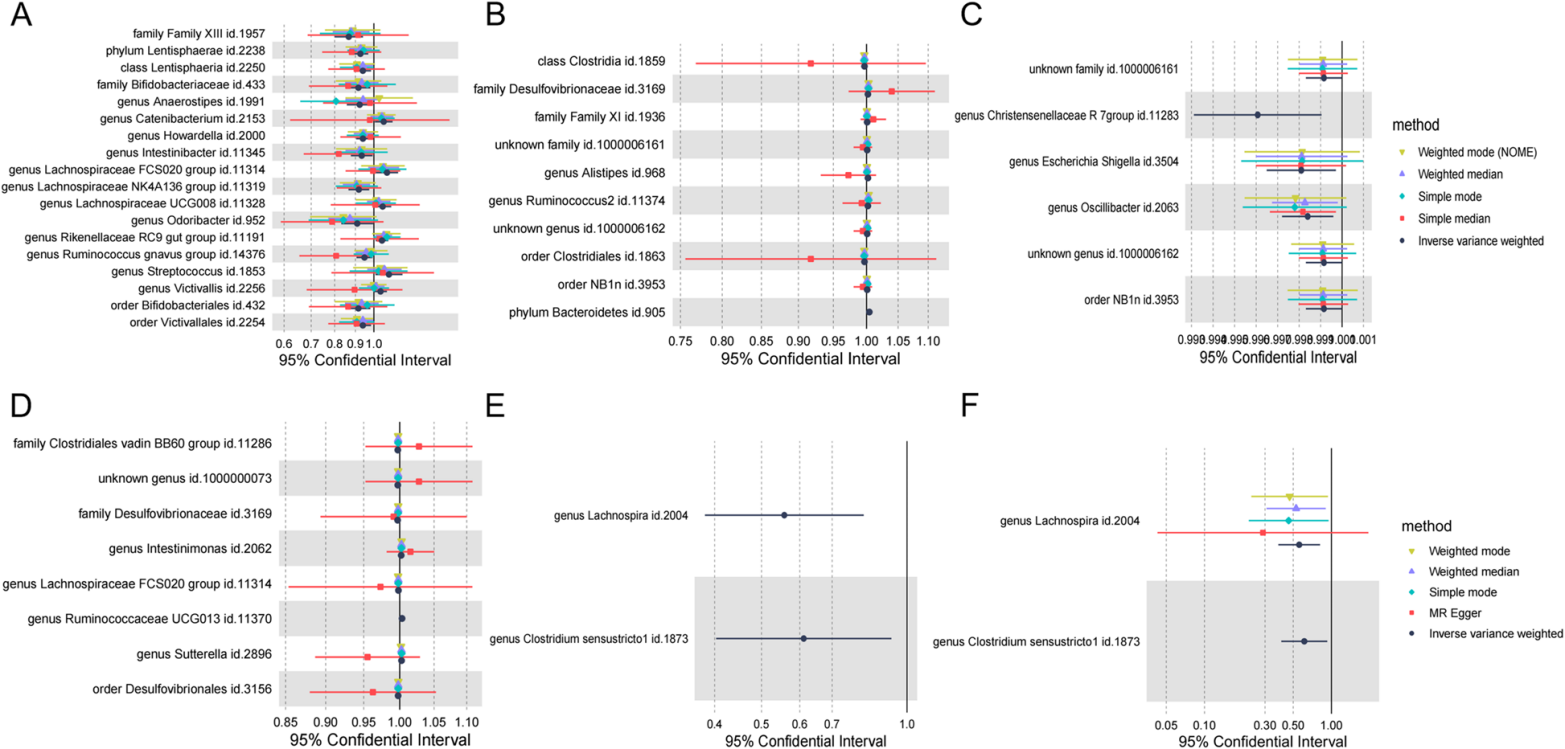
Causal relationship between gut flora and various types of cardiac arrhythmias. **A** Causal relationship between gut flora and atrial fibrillation. **B** Causal relationship between metabolites and supraventricular tachycardia. **C** Causal relationship between gut flora and tachycardia. **D** Causal relationship between gut flora and bradycardia. **E** Causal relationship between gut flora and atrioventricular block. **F** Causal relationship between gut flora and left bundle branch block

IVW analysis showed that family Family XIII id.1957 (OR = 0.87, 95% CI: 0.80-0.0.94, *p* = 0.0005) and phylum Lentisphaerae id.2238 (OR = 0.93, 95% CI: 0.89–0.97, *p* = 0.0007) and other 10 gut flora were negatively associated with the development of atrial fibrillation. genus Lachnospiraceae FCS020 group id.11314 (OR = 1.08, 95% CI: 1.01–1.15, *p* = 0.0209) and genus Streptococcus id.1853 (OR = 1.09, 95% CI: 1.01–1.18, *p* = 0.0299) and 4 other gut flora were positively associated with the development of atrial fibrillation (Supplementary Table S3) (Fig. 2A).

#### Causal relationship between gut flora and supraventricular tachycardia

IVW analysis showed that Family XIII id.1957 (OR = 0.99, 95% CI: 1.00–1.00, *p* = 0.0099) and phylum Lentisphaerae id.2238 (OR = 0.99, 95% CI: 0.99–1.00, *p* = 0.01659) and other 8 gut flora were positively associated with the development of supraventricular tachycardia (Supplementary Table S4) (Fig. 2B).

#### Causal relationship between gut flora and tachycardia

IVW analysis showed that 8 species of gut flora including family Desulfovibrionaceae id.3169 (OR = 0.99, 95% CI: 0.99–1.00, *p* = 0.0040) and genus Ruminococcaceae UCG013 id.11370 (OR = 1.01, 95% CI:1.00- 1.01, *p* = 0.0193) were positively associated with the development of tachycardia (Supplementary Table S5) (Fig. 2C).

#### Causal relationship between gut flora and bradycardia

IVW analysis showed that 6 gut flora including genus Christensenellaceae R 7group id.11283 (OR = 0.99, 95% CI: 0.99–0.99, *p* = 0.0096) and genus Escherichia Shigella id.3504 (OR = 1.00, 95% CI. 1.00–1.00, *p* = 0.0210) were positively associated with the development of bradycardia (Supplementary Table S6) (Fig. 2D).

#### Causal relationship between gut flora and atrioventricular block

IVW analysis showed that 2 gut flora, including genus Lachnospira id.2004 (OR = 0.56, 95% CI: 0.38–0.81, *p* = 0.0024) and genus Clostridium sensustricto1 id.1873 (OR = 0.61, 95% CI: 0.40–0.93, *p* = 0.0210) (Supplementary Table S7) (Fig. 2E).

At the same time, we found that the causal relationship between gut flora and the organization of left bundle branch block and atrioventricular block obtained by our analysis was almost the same(Supplementary Table S8) (Fig. 2F).

### Causal relationship between metabolites and various types of arrhythmias

#### Causal relationship between metabolites and atrial fibrillation

**Fig. 3:**
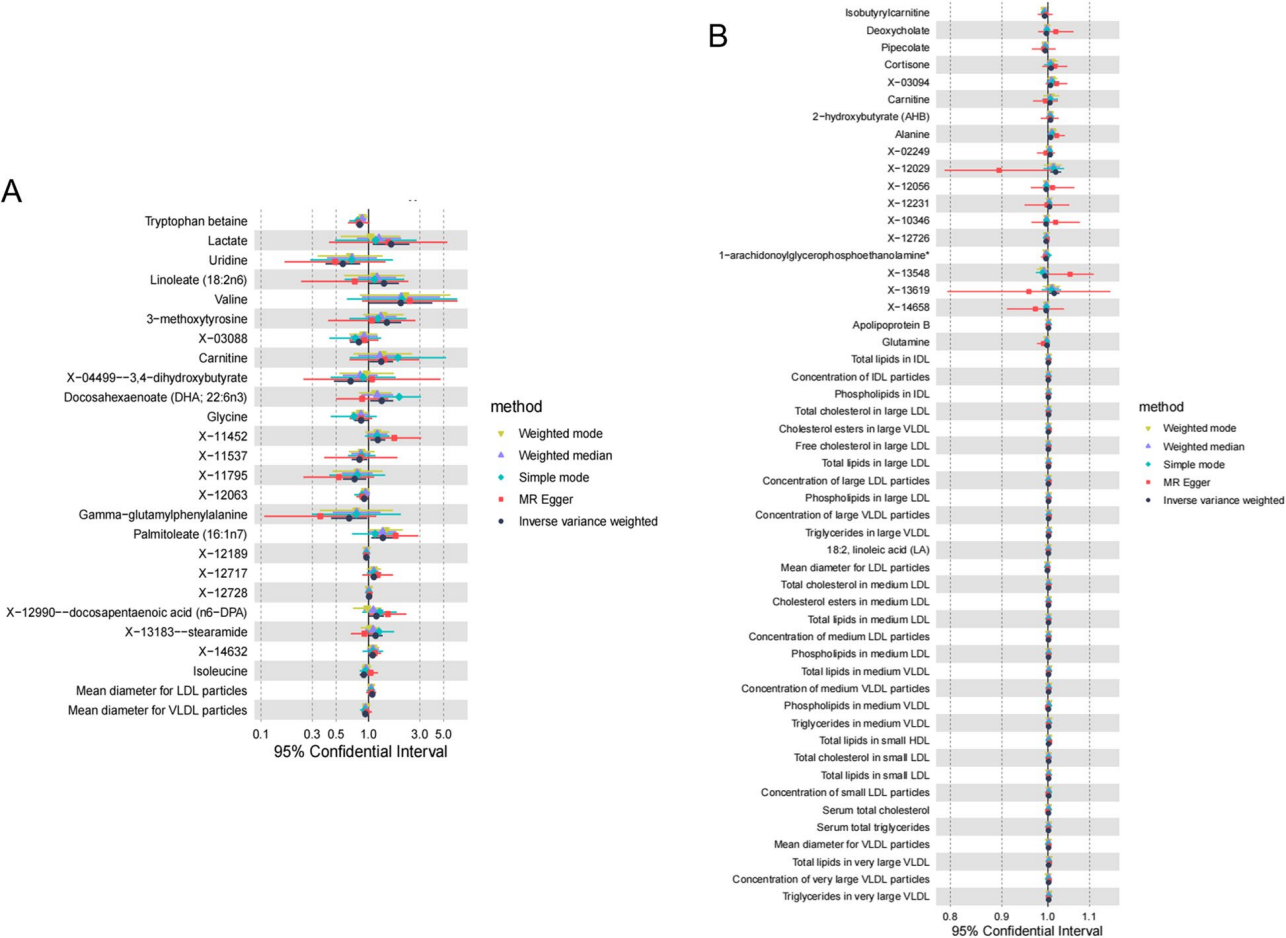
Causal relationship between metabolites and atrial fibrillation and supraventricular tachycardia. **A** Causal relationship between metabolites and atrial fibrillation. **B** Causal relationship between metabolites and supraventricular tachycardia

IVW analysis showed that 12 metabolites, including Tryptophan betaine (OR = 0.83, 95% CI: 0.76–0.90, *p* = 0.0001) and Uridine (OR = 0.58, 95% CI: 0.40–0.84, *p* = 0.0037) were negatively associated with the development of AF. 14 metabolites including Docosahexaenoate (DHA; 22:6n3) (OR = 1.33, 95% CI:1.04–1.70, *p* = 0.0252) and Carnitine (OR = 1.31, 95% CI:1.02–1.69, *p* = 0.0348), were positively associated with the onset of AF (Supplementary Table S9) (Fig. 3A).

#### Causal relationship between metabolites and supraventricular tachycardia

IVW analysis showed that 3 metabolites, Isobutyrylcarnitine (OR = 0.99, 95% CI: 0.99–1.00, *p* = 0.0003) and Pipecolate (OR = 0.99, 95% CI: 0.99–1.00, *p* = 0.0275) were negatively associated with the development of supraventricular tachycardia. Triglycerides in large VLDL (OR = 1.33, 95% CI: 1.00–1.00, *p* = 0.0131) and total cholesterol in small LDL (OR = 1.00, 95% CI: 1.00–1.00, *p* = 0.0016) and 47 other metabolites were positively associated with the development of supraventricular tachycardia (Supplementary Table S10) (Fig. 3B).

#### Causal relationship between metabolites and tachycardia

**Fig. 4:**
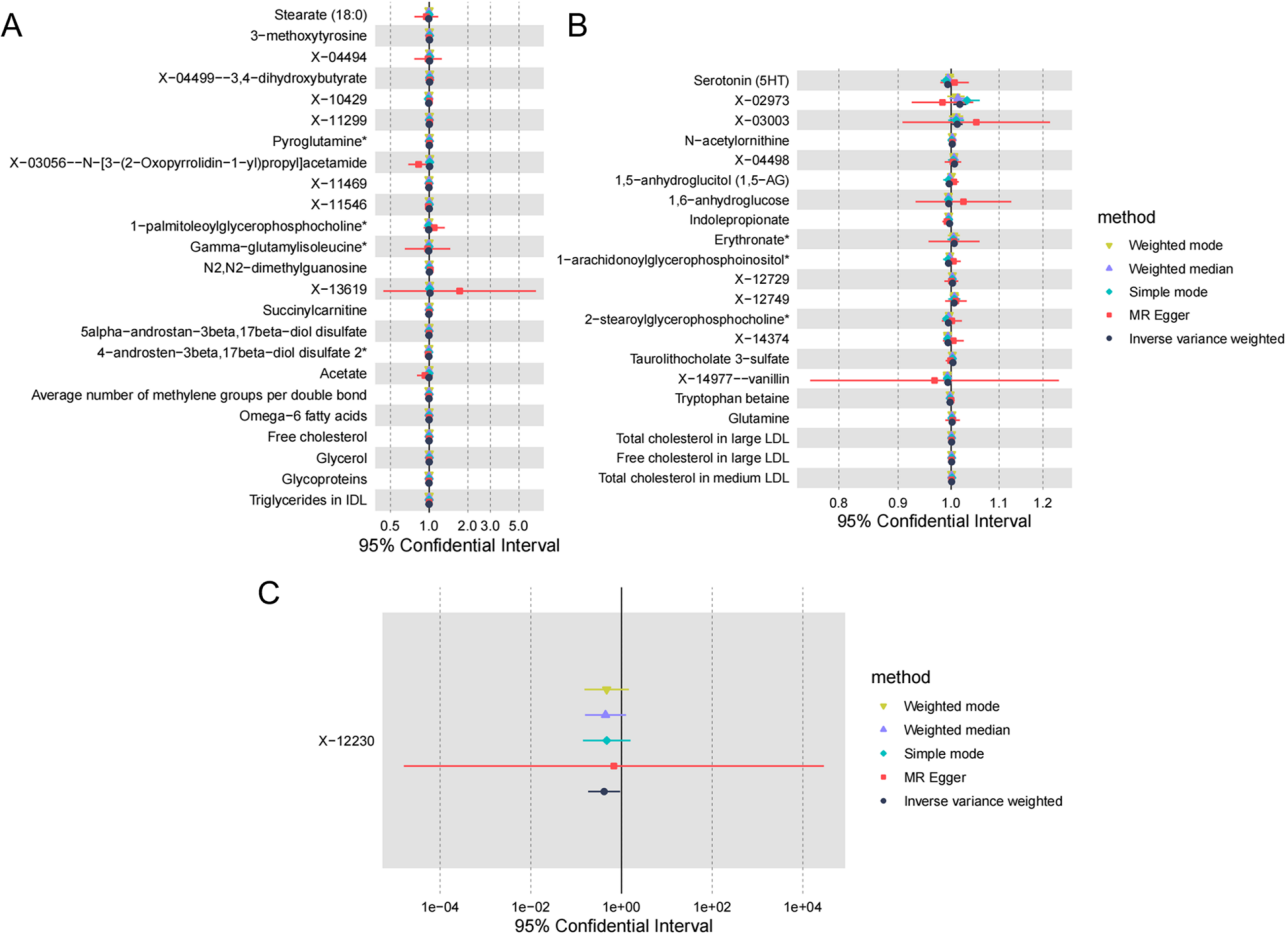
Causal relationship between metabolites and tachycardia, bradycardia and atrioventricular block. **A** Causal relationship between metabolites and tachycardia. **B** Causal relationship between metabolites and bradycardia. **C** Causal relationship between metabolites and atrioventricular block

IVW analysis showed that 4 metabolites, 1-palmitoleoylglycerophosphocholine (OR = 0.99, 95% CI:0.99–1.00, *p* = 0.0217) and gamma-glutamylisoleucine (OR = 0.99, 95% CI:0.98–1.00, *p* = 0.0477) were negatively associated with the onset of tachycardia. N2-dimethylguanosine (OR = 1.01, 95% CI:1.00–1.00, *p* = 0.0376) and X-04499–3,4-dihydroxybutyrate (OR = 1.01, 95% CI: 1.00–1.02, *p* = 0.0127) and 19 other metabolites were positively associated with the development of tachycardia (Supplementary Table S11) (Fig. 4A).

#### Causal relationship between metabolites and bradycardia

IVW analysis showed that 4 metabolites, 2-stearoylglycerophosphocholine (OR = 0.99, 95% CI:0.99–1.00, *p* = 0.0051) and Serotonin (5HT) (OR = 0.99, 95% CI:0.99–1.00, *p* = 0.0072) were negatively associated with the onset of bradycardia. Erythronate (OR = 1.01, 95% CI: 1.00–1.01, *p* = 0.0438) and 1-arachidonoylglycerophosphoinositol (OR = 1.01, 95% CI: 1.00–1.01, *p* = 0.0471) and 15 other metabolites were positively associated with the development of tachycardia (Supplementary Table S12) (Fig. 4B).

#### Causal relationship between metabolites and atrioventricular block

IVW analysis showed that X-12230 (OR = 0.41, 95% CI: 0.18–0.93, *p* = 0.0338) was negatively associated with the onset of atrioventricular block, and no metabolite was positively associated with the onset of atrioventricular block (Supplementary Table S13) (Fig. 4C).

### Sensitivity analysis

Heterogeneity test: the results of the Q-test showed no heterogeneity between the included SNPs (*p* > 0.05). Horizontal pleiotropy: The results of MR-Egger regression intercepts showed no horizontal pleiotropy in the associations of gut flora and serum metabolites with the associations with each type of arrhythmia. The absence of SNPs with large effects on effect estimates in the analyses of gut flora and serum metabolites with each type of arrhythmia suggests that a causal relationship exists and that the causal relationship is reasonably stable.

## Discussion

There is already a lot of research supporting the theory of a "gut-heart" axis-centred relationship between gut microbes and heart health, which means that the gut flora can influence the host's metabolism, inflammation levels, and immune system, which ultimately affects the heart's health [24–26]. Based on the analyses in this study, we have also identified a variety of gut flora and serum metabolites that may be closely related to inflammatory responses and immune regulation.

Interactions between inflammatory responses, cardiac structural remodeling, and electrophysiological remodeling are significant contributors to various arrhythmias [27, 28]. This study found a negative association between various gut flora, including Bifidobacteriales, Odoribacter, and Ruminococcaceae, and the risk of developing atrial fibrillation. Some of these flora are also implicated in key aspects of the inflammatory response. For example, Bifidobacterium bifidum has been shown to reduce the risk of atherosclerosis in ex vivo experiments, with one of the mechanisms being alteration of cholesterol metabolism, reduction of oxidative stress, and lowering of trimethylamine oxide (TMAO) levels [29–31]. Another study found that the abundance of Bifidobacterium and Ruminococcaceae were inversely related to different markers of low-grade inflammation such as hsCRP and interleukin (IL)-6, and Ruminococcaceae ruminantium reduces the inflammatory response by modulating T cell numbers and producing short-chain fatty acids [32, 33]. Therefore, we hypothesised that intestinal flora such as Bifidobacterium and Ruminococcaceae may reduce the risk of AF by suppressing the inflammatory response. Our study identified Clostridium lachnospira and Clostridium sensustricto, bacteria known for their ability to break down carbohydrates. Chronic consumption of high-sugar carbohydrates may lead to insulin resistance, which is associated with the development of chronic inflammation, and therefore we hypothesise that these two bacteria may influence the risk of developing left bundle branch block and atrioventricular block through this mechanism. Additionally, our study identified anaerobic bacteria like Lachnospira and Rikenellaceae, which may be linked to an increased risk of arrhythmia These anaerobic bacteria break down carbohydrates and produce short-chain fatty acids (e.g., acetic acid, propionic acid, and butyric acid) and alcohols (ethanol, isopropanol, and butanol), which affect the activity of immune cells and inflammatory responses, and the various alcohols they produce are metabolised by alcohol, causing cardiomyocyte damage and death, which ultimately leads to alterations in cardiac structure and function [34, 35].

Cardiomyocytes that are chronically stimulated by these substances may greatly increase the risk of developing arrhythmias, which can eventually lead to heart failure and, in severe cases, sudden death [34, 35]. The alteration of ion channels on the membrane surface of cardiomyocytes is called electrophysiological remodelling. It has been suggested that dysbiosis of the intestinal flora and altered levels of TMAO may both be closely related to electrophysiological remodelling [30, 36, 37]. Combined with our findings that flora such as Ruminococcaceae, which is associated with the risk of developing supraventricular tachycardia and atrial fibrillation, is able to convert dietary choline, lecithin and L-carnitine into trimethylamine, which is then further oxidised in the liver to TMAO, this could therefore be one of the reasons why intestinal flora can contribute to cardiac arrhythmias [38, 39]. Immunocytes such as macrophages are normally present in large numbers right in the heart, and the inflammatory response can regulate calcium homeostasis and connexins through pathways such as CXCR4 and TYROBP and cause changes in atrial electrophysiology and structural substrates, as well as affecting the resting membrane potential and action potential of cardiomyocytes [40–42]. Flora such as Bifidobacterium and Lactobacillus were identified in this study, which have been shown to be involved in modulating the immune response and may therefore influence the risk of arrhythmia development from this mechanism [43, 44].

Subsequently, we co-analysed the MR results of serum metabolomics with those of intestinal flora and found similarities between the trends of some intestinal flora and those of serum metabolites, which may collectively affect the risk of arrhythmia development. The heart is controlled by the autonomic nervous system, and a variety of neurotransmitters affect the electrophysiological properties of cardiac cells, including the duration and conduction velocity of action potentials. The present study found that serum metabolites such as tryptophan, isoleucine and valine may be closely associated with the risk of AF. Branched-chain amino acids such as tryptophan are one of the raw materials for synthesising neurotransmitters and it has been demonstrated that there is a close correlation between the relationship between elevated branched-chain amino acids and cardiac arrhythmias, so our study further provides some theoretical support for this conclusion [45]. At the same time, metabolites such as tryptophan are intricately linked to inflammatory regulation and immune modulation, as part of the metabolism of tryptophan takes place in the gut [46]. In the results of previous MR gut flora analyses, Bifidobacteria, Anaerobacteria, and Odorobacteria were found to be potentially protective against certain types of cardiac arrhythmias, and all of these flora were shown to have a strong relationship with tryptophan metabolism [40]. In addition, our analyses revealed that uridine may be strongly associated with the risk of developing AF. It has been suggested that uridine may modulate the inflammatory response by inhibiting inflammatory cell activity and other pathways, but whether it affects the risk of developing AF through this pathway deserves further exploration [47, 48]. Lactate and valine are both common serum metabolites that were similarly found in this study to potentially influence the risk of developing atrial fibrillation. It has been demonstrated that lactate accelerates vascular calcification and leads to mitochondrial dysfunction, and that valine exhibits antiarrhythmic effects in ischaemia–reperfusion experiments, mechanisms of action that may be closely related to cardiomyocyte apoptosis and inflammatory responses [49–51]. Meanwhile, combined with the results of our intestinal flora MR analyses, it was demonstrated that flora such as Bactericide and genus Odoribacter are strongly associated with lactate and valine, but the mechanism of action in disease remains unclear [52]. In addition, we have identified serum metabolites that may be associated with the risk of developing supraventricular tachycardia, bradycardia, tachypnea, and atrioventricular block, but the effects may not be as closely related as those of atrial fibrillation.

## Prospects and limitations of clinical applications

Based on the aforementioned research findings, the potential roles of gut microbiota and serum metabolites in arrhythmias reveal promising prospects for future clinical applications. Initially, alterations in specific gut microbiota and serum metabolites may serve as biomarkers for the early detection of arrhythmias. Furthermore, understanding how particular gut microbiota influence arrhythmias can aid in the development of personalized treatment plans. For instance, modifying one's diet to alter microbial composition, such as increasing probiotics, prebiotics, or certain types of fiber, might reduce the risk of atrial fibrillation if an individual's mouth and gut flora indicates a higher predisposition [53, 54]. Additionally, since gut microbiota can affect inflammation and immune responses through various mechanisms, they might present novel therapeutic targets for arrhythmia management. Despite this field offering many promising directions, current research is primarily still in the exploratory stage.

Limitations of this study include: Firstly, the research data encompasses only subjects of European descent, potentially limiting applicability to other populations. Secondly, although this is the first use of MR to investigate potential causal relationships between gut microbiota, metabolomics, and various types of arrhythmias, most conclusions are still theoretical. Further extensive research is required to establish specific causal links, evaluate intervention effectiveness, and consider inter-individual differences. Lastly, some gut microbiota and serum metabolites are still in the naming phase, with specific bacteria not yet definitively identified.

## Conclusion

Gut flora and serum metabolites may influence the risk of developing arrhythmias, but finding key gut flora and serum metabolites and exploring their specific mechanisms still requires extensive experimental validation.

### Supplementary Information

**Additional file 1: Supplementary Table 1. **2,232 SNPs in the gut flora were identified as Final IV.

**Additional file 2: Supplementary table 2. **10,075 SNPs in serum metabolites were identified as Final IV.

**Additional file 3: Supplementary Table S3. **Causal relationship between gut flora and atrial fibrillation.

**Additional file 4: Supplementary Table S4. **Causal relationship between gut flora and supraventricular.

**Additional file 5: Supplementary Table S5. **Causal relationship between gut flora and tachycardia.

**Additional file 6: Supplementary Table S6. **Causal relationship between gut flora and bradycardia.

**Additional file 7: Supplementary Table S7. **Causal relationship between gut flora and atrioventricular block.

**Additional file 8: Supplementary Table S8. **Causal relationship between gut flora and left bundle branch block.

**Additional file 9: Supplementary Table S9. **Causal relationship between metabolites and atrial fibrillation.

**Additional file 10: Supplementary Table S10. **Causal relationship between metabolites and supraventricular tachycardia.

**Additional file 11: Supplementary Table S11. **Causal relationship between metabolites and tachycardia.

**Additional file 12: Supplementary Table S12. **Causal relationship between metabolites and bradycardia.

**Additional file 13: Supplementary Table S13. **Causal relationship between metabolites and atrioventricular block.

## Data Availability

Publicly available datasets were analyzed in this study. This data can be found at: https://mibiogen.gcc.rug.nl/, https://r7.finngen.fi/, and https://gwas.mrcieu.ac.uk./
