# Supplementary material for: Putative causal relations among gut flora, serums metabolites and arrhythmia: a Mendelian randomization study
Source: BMC Cardiovasc Disord. 2024 Jan 11;24:38. doi: 10.1186/s12872-023-03703-z (PMC10782588; doi:10.1186/s12872-023-03703-z)
Supplement: Supplementary file 3 — Additional file 3: Supplementary Table S3. Causal relationship between gut flora and atrial fibrillation. [file 12872_2023_3703_MOESM3_ESM.docx]

**Supplementary Table S3. Causal relationship between gut flora and atrial fibrillation**

|  | **Exposure（Bacterial traits）** | **Methods** | **N.SNP** | ***P*.val** | **OR** | **95% CI-**  **lower** | **95% CI-**  **upper** |
| --- | --- | --- | --- | --- | --- | --- | --- |
| Atrial fibrillation \|\| id:ebi-a-GCST006414 | family Family XIII id.1957 | Inverse variance weighted | 11.00 | 0.0005 | 0.87 | 0.80 | 0.94 |
| Atrial fibrillation \|\| id:ebi-a-GCST006414 | phylum Lentisphaerae id.2238 | Inverse variance weighted | 9.00 | 0.0007 | 0.93 | 0.89 | 0.97 |
| Atrial fibrillation \|\| id:ebi-a-GCST006414 | genus Howardella id.2000 | Inverse variance weighted | 10.00 | 0.0017 | 0.94 | 0.90 | 0.98 |
| Atrial fibrillation \|\| id:ebi-a-GCST006414 | genus Lachnospiraceae NK4A136 group id.11319 | Inverse variance weighted | 15.00 | 0.0043 | 0.92 | 0.87 | 0.97 |
| Atrial fibrillation \|\| id:ebi-a-GCST006414 | class Lentisphaeria id.2250 | Inverse variance weighted | 8.00 | 0.0084 | 0.94 | 0.90 | 0.98 |
| Atrial fibrillation \|\| id:ebi-a-GCST006414 | order Victivallales id.2254 | Inverse variance weighted | 8.00 | 0.0084 | 0.94 | 0.90 | 0.98 |
| Atrial fibrillation \|\| id:ebi-a-GCST006414 | family Bifidobacteriaceae id.433 | Inverse variance weighted | 11.00 | 0.0115 | 0.92 | 0.86 | 0.98 |
| Atrial fibrillation \|\| id:ebi-a-GCST006414 | order Bifidobacteriales id.432 | Inverse variance weighted | 11.00 | 0.0115 | 0.92 | 0.86 | 0.98 |
| Atrial fibrillation \|\| id:ebi-a-GCST006414 | genus Rikenellaceae RC9 gut group id.11191 | Inverse variance weighted | 11.00 | 0.0123 | 1.05 | 1.01 | 1.09 |
| Atrial fibrillation \|\| id:ebi-a-GCST006414 | genus Lachnospiraceae FCS020 group id.11314 | Inverse variance weighted | 12.00 | 0.0209 | 1.08 | 1.01 | 1.15 |
| Atrial fibrillation \|\| id:ebi-a-GCST006414 | genus Ruminococcus gnavus group id.14376 | Inverse variance weighted | 12.00 | 0.0218 | 0.95 | 0.91 | 0.99 |
| Atrial fibrillation \|\| id:ebi-a-GCST006414 | genus Intestinibacter id.11345 | Inverse variance weighted | 15.00 | 0.0282 | 0.93 | 0.88 | 0.99 |
| Atrial fibrillation \|\| id:ebi-a-GCST006414 | genus Streptococcus id.1853 | Inverse variance weighted | 15.00 | 0.0299 | 1.09 | 1.01 | 1.18 |
| Atrial fibrillation \|\| id:ebi-a-GCST006414 | genus Catenibacterium id.2153 | Inverse variance weighted | 5.00 | 0.0303 | 1.06 | 1.01 | 1.11 |
| Atrial fibrillation \|\| id:ebi-a-GCST006414 | genus Anaerostipes id.1991 | Inverse variance weighted | 13.00 | 0.0309 | 0.92 | 0.86 | 0.99 |
| Atrial fibrillation \|\| id:ebi-a-GCST006414 | genus Odoribacter id.952 | Inverse variance weighted | 7.00 | 0.0401 | 0.91 | 0.83 | 1.00 |
| Atrial fibrillation \|\| id:ebi-a-GCST006414 | genus Victivallis id.2256 | Inverse variance weighted | 10.00 | 0.0437 | 1.04 | 1.00 | 1.08 |
| Atrial fibrillation \|\| id:ebi-a-GCST006414 | genus Lachnospiraceae UCG008 id.11328 | Inverse variance weighted | 11.00 | 0.0472 | 1.05 | 1.00 | 1.10 |
