# Supplementary material for: Putative causal relations among gut flora, serums metabolites and arrhythmia: a Mendelian randomization study
Source: BMC Cardiovasc Disord. 2024 Jan 11;24:38. doi: 10.1186/s12872-023-03703-z (PMC10782588; doi:10.1186/s12872-023-03703-z)
Supplement: Supplementary file 4 — Additional file 4: Supplementary Table S4. Causal relationship between gut flora and supraventricular. [file 12872_2023_3703_MOESM4_ESM.docx]

**Supplementary Table S4. Causal relationship between gut flora and supraventricular tachycardia**

| **Outcome** | **Exposure（Bacterial traits）** | **Methods** | **N.SNP** | ***P*.val** | **OR** | **95% CI-**  **lower** | **95% CI-**  **upper** |
| --- | --- | --- | --- | --- | --- | --- | --- |
| Diagnoses - main ICD10: I47.1 Supraventricular tachycardia \|\| id:ukb-b-11748 | genus Alistipes id.968 | Inverse variance weighted | 7 | 0.0099 | 1.00 | 1.00 | 1.00 |
| Diagnoses - main ICD10: I47.1 Supraventricular tachycardia \|\| id:ukb-b-11748 | class Clostridia id.1859 | Inverse variance weighted | 3 | 0.0165 | 1.00 | 0.99 | 1.00 |
| Diagnoses - main ICD10: I47.1 Supraventricular tachycardia \|\| id:ukb-b-11748 | order Clostridiales id.1863 | Inverse variance weighted | 3 | 0.0166 | 1.00 | 0.99 | 1.00 |
| Diagnoses - main ICD10: I47.1 Supraventricular tachycardia \|\| id:ukb-b-11748 | unknown family id.1000006161 | Inverse variance weighted | 8 | 0.0184 | 1.00 | 1.00 | 1.00 |
| Diagnoses - main ICD10: I47.1 Supraventricular tachycardia \|\| id:ukb-b-11748 | unknown genus id.1000006162 | Inverse variance weighted | 8 | 0.0184 | 1.00 | 1.00 | 1.00 |
| Diagnoses - main ICD10: I47.1 Supraventricular tachycardia \|\| id:ukb-b-11748 | order NB1n id.3953 | Inverse variance weighted | 8 | 0.0184 | 1.00 | 1.00 | 1.00 |
| Diagnoses - main ICD10: I47.1 Supraventricular tachycardia \|\| id:ukb-b-11748 | genus Ruminococcus2 id.11374 | Inverse variance weighted | 5 | 0.0256 | 1.00 | 1.00 | 1.00 |
| Diagnoses - main ICD10: I47.1 Supraventricular tachycardia \|\| id:ukb-b-11748 | family Family XI id.1936 | Inverse variance weighted | 5 | 0.0279 | 1.00 | 1.00 | 1.00 |
| Diagnoses - main ICD10: I47.1 Supraventricular tachycardia \|\| id:ukb-b-11748 | phylum Bacteroidetes id.905 | Inverse variance weighted | 2 | 0.0292 | 1.00 | 1.00 | 1.01 |
| Diagnoses - main ICD10: I47.1 Supraventricular tachycardia \|\| id:ukb-b-11748 | family Desulfovibrionaceae id.3169 | Inverse variance weighted | 4 | 0.0409 | 1.00 | 1.00 | 1.00 |
