# Supplementary material for: Putative causal relations among gut flora, serums metabolites and arrhythmia: a Mendelian randomization study
Source: BMC Cardiovasc Disord. 2024 Jan 11;24:38. doi: 10.1186/s12872-023-03703-z (PMC10782588; doi:10.1186/s12872-023-03703-z)
Supplement: Supplementary file 5 — Additional file 5: Supplementary Table S5. Causal relationship between gut flora and tachycardia. [file 12872_2023_3703_MOESM5_ESM.docx]

**Supplementary Table S5. Causal relationship between gut flora and tachycardia**

|  | **Exposure（Bacterial traits）** | **Methods** | **N.SNP** | ***P*.val** | **OR** | **95% CI-**  **lower** | **95% CI-**  **upper** |
| --- | --- | --- | --- | --- | --- | --- | --- |
| Diagnoses - secondary ICD10: R00.0 Tachycardia, unspecified \|\| id:ukb-b-17309 | family Clostridiales vadin BB60 group id.11286 | Inverse variance weighted | 3 | 0.0014 | 1.00 | 1.00 | 1.00 |
| Diagnoses - secondary ICD10: R00.0 Tachycardia, unspecified \|\| id:ukb-b-17309 | unknown genus id.1000000073 | Inverse variance weighted | 3 | 0.0014 | 1.00 | 1.00 | 1.00 |
| Diagnoses - secondary ICD10: R00.0 Tachycardia, unspecified \|\| id:ukb-b-17309 | genus Sutterella id.2896 | Inverse variance weighted | 4 | 0.0026 | 1.00 | 1.00 | 1.00 |
| Diagnoses - secondary ICD10: R00.0 Tachycardia, unspecified \|\| id:ukb-b-17309 | family Desulfovibrionaceae id.3169 | Inverse variance weighted | 3 | 0.0040 | 1.00 | 0.99 | 1.00 |
| Diagnoses - secondary ICD10: R00.0 Tachycardia, unspecified \|\| id:ukb-b-17309 | order Desulfovibrionales id.3156 | Inverse variance weighted | 4 | 0.0166 | 1.00 | 1.00 | 1.00 |
| Diagnoses - secondary ICD10: R00.0 Tachycardia, unspecified \|\| id:ukb-b-17309 | genus Ruminococcaceae UCG013 id.11370 | Inverse variance weighted | 2 | 0.0193 | 1.00 | 1.00 | 1.01 |
| Diagnoses - secondary ICD10: R00.0 Tachycardia, unspecified \|\| id:ukb-b-17309 | genus Intestinimonas id.2062 | Inverse variance weighted | 3 | 0.0230 | 1.00 | 1.00 | 1.00 |
| Diagnoses - secondary ICD10: R00.0 Tachycardia, unspecified \|\| id:ukb-b-17309 | genus Lachnospiraceae FCS020 group id.11314 | Inverse variance weighted | 4 | 0.0313 | 1.00 | 1.00 | 1.00 |
