# Supplementary material for: Putative causal relations among gut flora, serums metabolites and arrhythmia: a Mendelian randomization study
Source: BMC Cardiovasc Disord. 2024 Jan 11;24:38. doi: 10.1186/s12872-023-03703-z (PMC10782588; doi:10.1186/s12872-023-03703-z)
Supplement: Supplementary file 6 — Additional file 6: Supplementary Table S6. Causal relationship between gut flora and bradycardia. [file 12872_2023_3703_MOESM6_ESM.docx]

**Supplementary Table S6. Causal relationship between gut flora and bradycardia**

|  | **Exposure（Bacterial traits）** | **Methods** | **N.SNP** | ***P*.val** | **OR** | **95% CI-**  **lower** | **95% CI-**  **upper** |
| --- | --- | --- | --- | --- | --- | --- | --- |
| Diagnoses - secondary ICD10: R00.1 Bradycardia, unspecified \|\| id:ukb-b-11664 | genus Oscillibacter id.2063 | Inverse variance weighted | 6 | 0.0086 | 1.00 | 1.00 | 1.00 |
| Diagnoses - secondary ICD10: R00.1 Bradycardia, unspecified \|\| id:ukb-b-11664 | genus Christensenellaceae R 7group id.11283 | Inverse variance weighted | 2 | 0.0096 | 1.00 | 0.99 | 1.00 |
| Diagnoses - secondary ICD10: R00.1 Bradycardia, unspecified \|\| id:ukb-b-11664 | genus Escherichia Shigella id.3504 | Inverse variance weighted | 5 | 0.0210 | 1.00 | 1.00 | 1.00 |
| Diagnoses - secondary ICD10: R00.1 Bradycardia, unspecified \|\| id:ukb-b-11664 | unknown family id.1000006161 | Inverse variance weighted | 7 | 0.0491 | 1.00 | 1.00 | 1.00 |
| Diagnoses - secondary ICD10: R00.1 Bradycardia, unspecified \|\| id:ukb-b-11664 | unknown genus id.1000006162 | Inverse variance weighted | 7 | 0.0491 | 1.00 | 1.00 | 1.00 |
| Diagnoses - secondary ICD10: R00.1 Bradycardia, unspecified \|\| id:ukb-b-11664 | order NB1n id.3953 | Inverse variance weighted | 7 | 0.0491 | 1.00 | 1.00 | 1.00 |
