# Supplementary material for: Putative causal relations among gut flora, serums metabolites and arrhythmia: a Mendelian randomization study
Source: BMC Cardiovasc Disord. 2024 Jan 11;24:38. doi: 10.1186/s12872-023-03703-z (PMC10782588; doi:10.1186/s12872-023-03703-z)
Supplement: Supplementary file 7 — Additional file 7: Supplementary Table S7. Causal relationship between gut flora and atrioventricular block. [file 12872_2023_3703_MOESM7_ESM.docx]

**Supplementary Table S7. Causal relationship between gut flora and atrioventricular block**

|  | **Exposure（Bacterial traits）** | **Methods** | **N.SNP** | ***P*.val** | **OR** | **95% CI-**  **lower** | **95% CI-**  **upper** |
| --- | --- | --- | --- | --- | --- | --- | --- |
| AVBLOCK | genus Lachnospira id.2004 | Inverse variance weighted | 5 | 0.0024 | 0.56 | 0.38 | 0.81 |
| AVBLOCK | genus Clostridium sensustricto1 id.1873 | Inverse variance weighted | 2 | 0.0210 | 0.61 | 0.40 | 0.93 |
