# Supplementary material for: Putative causal relations among gut flora, serums metabolites and arrhythmia: a Mendelian randomization study
Source: BMC Cardiovasc Disord. 2024 Jan 11;24:38. doi: 10.1186/s12872-023-03703-z (PMC10782588; doi:10.1186/s12872-023-03703-z)
Supplement: Supplementary file 9 — Additional file 9: Supplementary Table S9. Causal relationship between metabolites and atrial fibrillation. [file 12872_2023_3703_MOESM9_ESM.docx]

**Supplementary Table S9. Causal relationship between metabolites and atrial fibrillation**

|  | **Exposure（Bacterial traits）** | **Methods** | **N.SNP** | ***P*.val** | **OR** | **95% CI-**  **lower** | **95% CI-**  **upper** |
| --- | --- | --- | --- | --- | --- | --- | --- |
| Atrial fibrillation \|\| id:ebi-a-GCST006414 | Tryptophan betaine | Inverse variance weighted | 10 | 0.0001 | 0.83 | 0.76 | 0.90 |
| Atrial fibrillation \|\| id:ebi-a-GCST006414 | Mean diameter for LDL particles | Inverse variance weighted | 25 | 0.0004 | 1.08 | 1.04 | 1.13 |
| Atrial fibrillation \|\| id:ebi-a-GCST006414 | X-12189 | Inverse variance weighted | 20 | 0.0015 | 0.96 | 0.93 | 0.98 |
| Atrial fibrillation \|\| id:ebi-a-GCST006414 | Mean diameter for VLDL particles | Inverse variance weighted | 31 | 0.0019 | 0.93 | 0.90 | 0.98 |
| Atrial fibrillation \|\| id:ebi-a-GCST006414 | X-12717 | Inverse variance weighted | 9 | 0.0035 | 1.12 | 1.04 | 1.21 |
| Atrial fibrillation \|\| id:ebi-a-GCST006414 | Uridine | Inverse variance weighted | 20 | 0.0037 | 0.58 | 0.40 | 0.84 |
| Atrial fibrillation \|\| id:ebi-a-GCST006414 | 3-methoxytyrosine | Inverse variance weighted | 16 | 0.0122 | 1.48 | 1.09 | 2.01 |
| Atrial fibrillation \|\| id:ebi-a-GCST006414 | X-11452 | Inverse variance weighted | 6 | 0.0148 | 1.22 | 1.04 | 1.43 |
| Atrial fibrillation \|\| id:ebi-a-GCST006414 | Palmitoleate (16:1n7) | Inverse variance weighted | 9 | 0.0154 | 1.36 | 1.06 | 1.74 |
| Atrial fibrillation \|\| id:ebi-a-GCST006414 | Lactate | Inverse variance weighted | 13 | 0.0159 | 1.62 | 1.09 | 2.40 |
| Atrial fibrillation \|\| id:ebi-a-GCST006414 | X-11795 | Inverse variance weighted | 12 | 0.0195 | 0.74 | 0.58 | 0.95 |
| Atrial fibrillation \|\| id:ebi-a-GCST006414 | X-11537 | Inverse variance weighted | 6 | 0.0226 | 0.82 | 0.70 | 0.97 |
| Atrial fibrillation \|\| id:ebi-a-GCST006414 | Isoleucine | Inverse variance weighted | 27 | 0.0233 | 0.90 | 0.83 | 0.99 |
| Atrial fibrillation \|\| id:ebi-a-GCST006414 | X-12728 | Inverse variance weighted | 33 | 0.0247 | 1.01 | 1.00 | 1.02 |
| Atrial fibrillation \|\| id:ebi-a-GCST006414 | Docosahexaenoate (DHA; 22:6n3) | Inverse variance weighted | 6 | 0.0252 | 1.33 | 1.04 | 1.70 |
| Atrial fibrillation \|\| id:ebi-a-GCST006414 | X-12063 | Inverse variance weighted | 17 | 0.0308 | 0.91 | 0.84 | 0.99 |
| Atrial fibrillation \|\| id:ebi-a-GCST006414 | X-04499--3,4-dihydroxybutyrate | Inverse variance weighted | 21 | 0.0347 | 0.68 | 0.48 | 0.97 |
| Atrial fibrillation \|\| id:ebi-a-GCST006414 | X-03088 | Inverse variance weighted | 19 | 0.0347 | 0.81 | 0.67 | 0.99 |
| Atrial fibrillation \|\| id:ebi-a-GCST006414 | Carnitine | Inverse variance weighted | 129 | 0.0348 | 1.31 | 1.02 | 1.69 |
| Atrial fibrillation \|\| id:ebi-a-GCST006414 | Gamma-glutamylphenylalanine | Inverse variance weighted | 20 | 0.0349 | 0.66 | 0.45 | 0.97 |
| Atrial fibrillation \|\| id:ebi-a-GCST006414 | Glycine | Inverse variance weighted | 25 | 0.0371 | 0.85 | 0.73 | 0.99 |
| Atrial fibrillation \|\| id:ebi-a-GCST006414 | Linoleate (18:2n6) | Inverse variance weighted | 17 | 0.0421 | 1.39 | 1.01 | 1.92 |
| Atrial fibrillation \|\| id:ebi-a-GCST006414 | X-12990--docosapentaenoic acid (n6-DPA) | Inverse variance weighted | 12 | 0.0424 | 1.18 | 1.01 | 1.39 |
| Atrial fibrillation \|\| id:ebi-a-GCST006414 | Valine | Inverse variance weighted | 6 | 0.0456 | 1.99 | 1.01 | 3.92 |
| Atrial fibrillation \|\| id:ebi-a-GCST006414 | X-14632 | Inverse variance weighted | 18 | 0.0474 | 1.09 | 1.00 | 1.19 |
| Atrial fibrillation \|\| id:ebi-a-GCST006414 | X-13183--stearamide | Inverse variance weighted | 10 | 0.0475 | 1.16 | 1.00 | 1.35 |
