# Supplementary material for: Putative causal relations among gut flora, serums metabolites and arrhythmia: a Mendelian randomization study
Source: BMC Cardiovasc Disord. 2024 Jan 11;24:38. doi: 10.1186/s12872-023-03703-z (PMC10782588; doi:10.1186/s12872-023-03703-z)
Supplement: Supplementary file 10 — Additional file 10: Supplementary Table S10. Causal relationship between metabolites and supraventricular tachycardia. [file 12872_2023_3703_MOESM10_ESM.docx]

**Supplementary Table S10.** **Causal relationship between metabolites and supraventricular tachycardia**

|  | **Exposure（Bacterial traits）** | **Methods** | **N.SNP** | ***P*.val** | **OR** | **95% CI-**  **lower** | **95% CI-**  **upper** |
| --- | --- | --- | --- | --- | --- | --- | --- |
| Diagnoses - main ICD10: I47.1 Supraventricular tachycardia \|\| id:ukb-b-11748 | Isobutyrylcarnitine | Inverse variance weighted | 6 | 0.0003 | 0.99 | 0.99 | 1.00 |
| Diagnoses - main ICD10: I47.1 Supraventricular tachycardia \|\| id:ukb-b-11748 | Concentration of small LDL particles | Inverse variance weighted | 8 | 0.0010 | 1.00 | 1.00 | 1.00 |
| Diagnoses - main ICD10: I47.1 Supraventricular tachycardia \|\| id:ukb-b-11748 | Total cholesterol in small LDL | Inverse variance weighted | 8 | 0.0016 | 1.00 | 1.00 | 1.00 |
| Diagnoses - main ICD10: I47.1 Supraventricular tachycardia \|\| id:ukb-b-11748 | Total cholesterol in large LDL | Inverse variance weighted | 12 | 0.0030 | 1.00 | 1.00 | 1.00 |
| Diagnoses - main ICD10: I47.1 Supraventricular tachycardia \|\| id:ukb-b-11748 | Total lipids in small LDL | Inverse variance weighted | 8 | 0.0032 | 1.00 | 1.00 | 1.00 |
| Diagnoses - main ICD10: I47.1 Supraventricular tachycardia \|\| id:ukb-b-11748 | Triglycerides in medium VLDL | Inverse variance weighted | 9 | 0.0049 | 1.00 | 1.00 | 1.00 |
| Diagnoses - main ICD10: I47.1 Supraventricular tachycardia \|\| id:ukb-b-11748 | X-12029 | Inverse variance weighted | 7 | 0.0051 | 1.02 | 1.01 | 1.03 |
| Diagnoses - main ICD10: I47.1 Supraventricular tachycardia \|\| id:ukb-b-11748 | Mean diameter for LDL particles | Inverse variance weighted | 7 | 0.0076 | 1.00 | 1.00 | 1.00 |
| Diagnoses - main ICD10: I47.1 Supraventricular tachycardia \|\| id:ukb-b-11748 | Total lipids in medium LDL | Inverse variance weighted | 12 | 0.0083 | 1.00 | 1.00 | 1.00 |
| Diagnoses - main ICD10: I47.1 Supraventricular tachycardia \|\| id:ukb-b-11748 | Cholesterol esters in medium LDL | Inverse variance weighted | 12 | 0.0085 | 1.00 | 1.00 | 1.00 |
| Diagnoses - main ICD10: I47.1 Supraventricular tachycardia \|\| id:ukb-b-11748 | Total lipids in large LDL | Inverse variance weighted | 12 | 0.0110 | 1.00 | 1.00 | 1.00 |
| Diagnoses - main ICD10: I47.1 Supraventricular tachycardia \|\| id:ukb-b-11748 | X-10346 | Inverse variance weighted | 3 | 0.0116 | 1.00 | 0.99 | 1.00 |
| Diagnoses - main ICD10: I47.1 Supraventricular tachycardia \|\| id:ukb-b-11748 | X-12056 | Inverse variance weighted | 5 | 0.0121 | 1.00 | 0.99 | 1.00 |
| Diagnoses - main ICD10: I47.1 Supraventricular tachycardia \|\| id:ukb-b-11748 | Concentration of medium LDL particles | Inverse variance weighted | 12 | 0.0124 | 1.00 | 1.00 | 1.00 |
| Diagnoses - main ICD10: I47.1 Supraventricular tachycardia \|\| id:ukb-b-11748 | Triglycerides in very large VLDL | Inverse variance weighted | 6 | 0.0135 | 1.00 | 1.00 | 1.00 |
| Diagnoses - main ICD10: I47.1 Supraventricular tachycardia \|\| id:ukb-b-11748 | Apolipoprotein B | Inverse variance weighted | 11 | 0.0142 | 1.00 | 1.00 | 1.00 |
| Diagnoses - main ICD10: I47.1 Supraventricular tachycardia \|\| id:ukb-b-11748 | Total cholesterol in medium LDL | Inverse variance weighted | 11 | 0.0146 | 1.00 | 1.00 | 1.00 |
| Diagnoses - main ICD10: I47.1 Supraventricular tachycardia \|\| id:ukb-b-11748 | Phospholipids in large LDL | Inverse variance weighted | 12 | 0.0158 | 1.00 | 1.00 | 1.00 |
| Diagnoses - main ICD10: I47.1 Supraventricular tachycardia \|\| id:ukb-b-11748 | Phospholipids in IDL | Inverse variance weighted | 14 | 0.0175 | 1.00 | 1.00 | 1.00 |
| Diagnoses - main ICD10: I47.1 Supraventricular tachycardia \|\| id:ukb-b-11748 | Alanine | Inverse variance weighted | 20 | 0.0175 | 1.01 | 1.00 | 1.01 |
| Diagnoses - main ICD10: I47.1 Supraventricular tachycardia \|\| id:ukb-b-11748 | Concentration of medium VLDL particles | Inverse variance weighted | 7 | 0.0180 | 1.00 | 1.00 | 1.00 |
| Diagnoses - main ICD10: I47.1 Supraventricular tachycardia \|\| id:ukb-b-11748 | X-13619 | Inverse variance weighted | 6 | 0.0183 | 1.01 | 1.00 | 1.03 |
| Diagnoses - main ICD10: I47.1 Supraventricular tachycardia \|\| id:ukb-b-11748 | Total lipids in small HDL | Inverse variance weighted | 7 | 0.0186 | 1.00 | 1.00 | 1.00 |
| Diagnoses - main ICD10: I47.1 Supraventricular tachycardia \|\| id:ukb-b-11748 | Phospholipids in medium LDL | Inverse variance weighted | 11 | 0.0196 | 1.00 | 1.00 | 1.00 |
| Diagnoses - main ICD10: I47.1 Supraventricular tachycardia \|\| id:ukb-b-11748 | 2-hydroxybutyrate (AHB) | Inverse variance weighted | 6 | 0.0213 | 1.01 | 1.00 | 1.01 |
| Diagnoses - main ICD10: I47.1 Supraventricular tachycardia \|\| id:ukb-b-11748 | Free cholesterol in large LDL | Inverse variance weighted | 13 | 0.0226 | 1.00 | 1.00 | 1.00 |
| Diagnoses - main ICD10: I47.1 Supraventricular tachycardia \|\| id:ukb-b-11748 | Concentration of large VLDL particles | Inverse variance weighted | 7 | 0.0232 | 1.00 | 1.00 | 1.00 |
| Diagnoses - main ICD10: I47.1 Supraventricular tachycardia \|\| id:ukb-b-11748 | 18:2, linoleic acid (LA) | Inverse variance weighted | 7 | 0.0273 | 1.00 | 1.00 | 1.00 |
| Diagnoses - main ICD10: I47.1 Supraventricular tachycardia \|\| id:ukb-b-11748 | Pipecolate | Inverse variance weighted | 4 | 0.0275 | 0.99 | 0.99 | 1.00 |
| Diagnoses - main ICD10: I47.1 Supraventricular tachycardia \|\| id:ukb-b-11748 | Deoxycholate | Inverse variance weighted | 5 | 0.0277 | 1.00 | 0.99 | 1.00 |
| Diagnoses - main ICD10: I47.1 Supraventricular tachycardia \|\| id:ukb-b-11748 | Concentration of IDL particles | Inverse variance weighted | 15 | 0.0287 | 1.00 | 1.00 | 1.00 |
| Diagnoses - main ICD10: I47.1 Supraventricular tachycardia \|\| id:ukb-b-11748 | Cortisone | Inverse variance weighted | 17 | 0.0290 | 1.01 | 1.00 | 1.01 |
| Diagnoses - main ICD10: I47.1 Supraventricular tachycardia \|\| id:ukb-b-11748 | Serum total cholesterol | Inverse variance weighted | 14 | 0.0302 | 1.00 | 1.00 | 1.00 |
| Diagnoses - main ICD10: I47.1 Supraventricular tachycardia \|\| id:ukb-b-11748 | X-14658 | Inverse variance weighted | 4 | 0.0309 | 1.00 | 0.99 | 1.00 |
| Diagnoses - main ICD10: I47.1 Supraventricular tachycardia \|\| id:ukb-b-11748 | X-12726 | Inverse variance weighted | 6 | 0.0312 | 1.00 | 0.99 | 1.00 |
| Diagnoses - main ICD10: I47.1 Supraventricular tachycardia \|\| id:ukb-b-11748 | Carnitine | Inverse variance weighted | 121 | 0.0316 | 1.00 | 1.00 | 1.01 |
| Diagnoses - main ICD10: I47.1 Supraventricular tachycardia \|\| id:ukb-b-11748 | Serum total triglycerides | Inverse variance weighted | 9 | 0.0318 | 1.00 | 1.00 | 1.00 |
| Diagnoses - main ICD10: I47.1 Supraventricular tachycardia \|\| id:ukb-b-11748 | Concentration of large LDL particles | Inverse variance weighted | 12 | 0.0326 | 1.00 | 1.00 | 1.00 |
| Diagnoses - main ICD10: I47.1 Supraventricular tachycardia \|\| id:ukb-b-11748 | Total lipids in very large VLDL | Inverse variance weighted | 6 | 0.0329 | 1.00 | 1.00 | 1.00 |
| Diagnoses - main ICD10: I47.1 Supraventricular tachycardia \|\| id:ukb-b-11748 | Total lipids in medium VLDL | Inverse variance weighted | 9 | 0.0331 | 1.00 | 1.00 | 1.00 |
| Diagnoses - main ICD10: I47.1 Supraventricular tachycardia \|\| id:ukb-b-11748 | X-12231 | Inverse variance weighted | 7 | 0.0336 | 1.00 | 1.00 | 1.01 |
| Diagnoses - main ICD10: I47.1 Supraventricular tachycardia \|\| id:ukb-b-11748 | X-13548 | Inverse variance weighted | 18 | 0.0357 | 0.99 | 0.99 | 1.00 |
| Diagnoses - main ICD10: I47.1 Supraventricular tachycardia \|\| id:ukb-b-11748 | Total lipids in IDL | Inverse variance weighted | 15 | 0.0364 | 1.00 | 1.00 | 1.00 |
| Diagnoses - main ICD10: I47.1 Supraventricular tachycardia \|\| id:ukb-b-11748 | X-03094 | Inverse variance weighted | 17 | 0.0376 | 1.01 | 1.00 | 1.01 |
| Diagnoses - main ICD10: I47.1 Supraventricular tachycardia \|\| id:ukb-b-11748 | Mean diameter for VLDL particles | Inverse variance weighted | 5 | 0.0384 | 1.00 | 1.00 | 1.00 |
| Diagnoses - main ICD10: I47.1 Supraventricular tachycardia \|\| id:ukb-b-11748 | Concentration of very large VLDL particles | Inverse variance weighted | 5 | 0.0409 | 1.00 | 1.00 | 1.00 |
| Diagnoses - main ICD10: I47.1 Supraventricular tachycardia \|\| id:ukb-b-11748 | Phospholipids in medium VLDL | Inverse variance weighted | 7 | 0.0414 | 1.00 | 1.00 | 1.00 |
| Diagnoses - main ICD10: I47.1 Supraventricular tachycardia \|\| id:ukb-b-11748 | Triglycerides in large VLDL | Inverse variance weighted | 8 | 0.0416 | 1.00 | 1.00 | 1.00 |
| Diagnoses - main ICD10: I47.1 Supraventricular tachycardia \|\| id:ukb-b-11748 | X-02249 | Inverse variance weighted | 5 | 0.0466 | 1.01 | 1.00 | 1.01 |
| Diagnoses - main ICD10: I47.1 Supraventricular tachycardia \|\| id:ukb-b-11748 | Cholesterol esters in large VLDL | Inverse variance weighted | 13 | 0.0472 | 1.00 | 1.00 | 1.00 |
| Diagnoses - main ICD10: I47.1 Supraventricular tachycardia \|\| id:ukb-b-11748 | 1-arachidonoylglycerophosphoethanolamine* | Inverse variance weighted | 11 | 0.0474 | 1.00 | 0.99 | 1.00 |
| Diagnoses - main ICD10: I47.1 Supraventricular tachycardia \|\| id:ukb-b-11748 | Glutamine | Inverse variance weighted | 6 | 0.0495 | 1.00 | 1.00 | 1.00 |
