# Supplementary material for: Putative causal relations among gut flora, serums metabolites and arrhythmia: a Mendelian randomization study
Source: BMC Cardiovasc Disord. 2024 Jan 11;24:38. doi: 10.1186/s12872-023-03703-z (PMC10782588; doi:10.1186/s12872-023-03703-z)
Supplement: Supplementary file 11 — Additional file 11: Supplementary Table S11. Causal relationship between metabolites and tachycardia. [file 12872_2023_3703_MOESM11_ESM.docx]

**Supplementary Table S11.** **Causal relationship between metabolites and tachycardia**

|  | **Exposure（Bacterial traits）** | **Methods** | | **N.SNP** | ***P*.val** | **OR** | **95% CI-**  **lower** | **95% CI-**  **upper** |
| --- | --- | --- | --- | --- | --- | --- | --- | --- |
| Diagnoses - secondary ICD10: R00.0 Tachycardia, unspecified \|\| id:ukb-b-17309 | X-04499--3,4-dihydroxybutyrate | | Inverse variance weighted | 6 | 0.0127 | 1.01 | 1.00 | 1.02 |
| Diagnoses - secondary ICD10: R00.0 Tachycardia, unspecified \|\| id:ukb-b-17309 | X-11546 | | Inverse variance weighted | 3 | 0.0137 | 1.00 | 1.00 | 1.00 |
| Diagnoses - secondary ICD10: R00.0 Tachycardia, unspecified \|\| id:ukb-b-17309 | Glycoproteins | | Inverse variance weighted | 5 | 0.0145 | 1.00 | 1.00 | 1.00 |
| Diagnoses - secondary ICD10: R00.0 Tachycardia, unspecified \|\| id:ukb-b-17309 | Average number of methylene groups per double bond | | Inverse variance weighted | 3 | 0.0160 | 1.00 | 1.00 | 1.00 |
| Diagnoses - secondary ICD10: R00.0 Tachycardia, unspecified \|\| id:ukb-b-17309 | 5alpha-androstan-3beta,17beta-diol disulfate | | Inverse variance weighted | 6 | 0.0169 | 1.00 | 1.00 | 1.00 |
| Diagnoses - secondary ICD10: R00.0 Tachycardia, unspecified \|\| id:ukb-b-17309 | X-11469 | | Inverse variance weighted | 3 | 0.0179 | 1.00 | 0.99 | 1.00 |
| Diagnoses - secondary ICD10: R00.0 Tachycardia, unspecified \|\| id:ukb-b-17309 | Triglycerides in IDL | | Inverse variance weighted | 14 | 0.0191 | 1.00 | 1.00 | 1.00 |
| Diagnoses - secondary ICD10: R00.0 Tachycardia, unspecified \|\| id:ukb-b-17309 | Pyroglutamine* | | Inverse variance weighted | 4 | 0.0194 | 1.00 | 1.00 | 1.01 |
| Diagnoses - secondary ICD10: R00.0 Tachycardia, unspecified \|\| id:ukb-b-17309 | Omega-6 fatty acids | | Inverse variance weighted | 8 | 0.0216 | 1.00 | 1.00 | 1.00 |
| Diagnoses - secondary ICD10: R00.0 Tachycardia, unspecified \|\| id:ukb-b-17309 | 1-palmitoleoylglycerophosphocholine* | | Inverse variance weighted | 5 | 0.0217 | 0.99 | 0.99 | 1.00 |
| Diagnoses - secondary ICD10: R00.0 Tachycardia, unspecified \|\| id:ukb-b-17309 | 4-androsten-3beta,17beta-diol disulfate 2* | | Inverse variance weighted | 8 | 0.0235 | 1.00 | 0.99 | 1.00 |
| Diagnoses - secondary ICD10: R00.0 Tachycardia, unspecified \|\| id:ukb-b-17309 | X-11299 | | Inverse variance weighted | 4 | 0.0240 | 1.00 | 1.00 | 1.00 |
| Diagnoses - secondary ICD10: R00.0 Tachycardia, unspecified \|\| id:ukb-b-17309 | X-04494 | | Inverse variance weighted | 6 | 0.0252 | 1.01 | 1.00 | 1.01 |
| Diagnoses - secondary ICD10: R00.0 Tachycardia, unspecified \|\| id:ukb-b-17309 | Succinylcarnitine | | Inverse variance weighted | 24 | 0.0318 | 1.00 | 0.99 | 1.00 |
| Diagnoses - secondary ICD10: R00.0 Tachycardia, unspecified \|\| id:ukb-b-17309 | Glycerol | | Inverse variance weighted | 5 | 0.0344 | 1.00 | 1.00 | 1.00 |
| Diagnoses - secondary ICD10: R00.0 Tachycardia, unspecified \|\| id:ukb-b-17309 | N2,N2-dimethylguanosine | | Inverse variance weighted | 16 | 0.0376 | 1.01 | 1.00 | 1.01 |
| Diagnoses - secondary ICD10: R00.0 Tachycardia, unspecified \|\| id:ukb-b-17309 | Acetate | | Inverse variance weighted | 3 | 0.0383 | 1.00 | 0.99 | 1.00 |
| Diagnoses - secondary ICD10: R00.0 Tachycardia, unspecified \|\| id:ukb-b-17309 | X-03056--N-[3-(2-Oxopyrrolidin-1-yl)propyl]acetamide | | Inverse variance weighted | 6 | 0.0387 | 1.01 | 1.00 | 1.02 |
| Diagnoses - secondary ICD10: R00.0 Tachycardia, unspecified \|\| id:ukb-b-17309 | Free cholesterol | | Inverse variance weighted | 8 | 0.0410 | 1.00 | 1.00 | 1.00 |
| Diagnoses - secondary ICD10: R00.0 Tachycardia, unspecified \|\| id:ukb-b-17309 | 3-methoxytyrosine | | Inverse variance weighted | 3 | 0.0413 | 1.01 | 1.00 | 1.01 |
| Diagnoses - secondary ICD10: R00.0 Tachycardia, unspecified \|\| id:ukb-b-17309 | Stearate (18:0) | | Inverse variance weighted | 12 | 0.0416 | 0.99 | 0.99 | 1.00 |
| Diagnoses - secondary ICD10: R00.0 Tachycardia, unspecified \|\| id:ukb-b-17309 | X-10429 | | Inverse variance weighted | 4 | 0.0436 | 0.99 | 0.99 | 1.00 |
| Diagnoses - secondary ICD10: R00.0 Tachycardia, unspecified \|\| id:ukb-b-17309 | Gamma-glutamylisoleucine* | | Inverse variance weighted | 7 | 0.0477 | 0.99 | 0.98 | 1.00 |
| Diagnoses - secondary ICD10: R00.0 Tachycardia, unspecified \|\| id:ukb-b-17309 | X-13619 | | Inverse variance weighted | 3 | 0.0487 | 1.02 | 1.00 | 1.03 |
| Diagnoses - secondary ICD10: R00.0 Tachycardia, unspecified \|\| id:ukb-b-17309 | X-04499--3,4-dihydroxybutyrate | | Inverse variance weighted | 6 | 0.0127 | 1.01 | 1.00 | 1.02 |
