# Supplementary material for: Putative causal relations among gut flora, serums metabolites and arrhythmia: a Mendelian randomization study
Source: BMC Cardiovasc Disord. 2024 Jan 11;24:38. doi: 10.1186/s12872-023-03703-z (PMC10782588; doi:10.1186/s12872-023-03703-z)
Supplement: Supplementary file 12 — Additional file 12: Supplementary Table S12. Causal relationship between metabolites and bradycardia. [file 12872_2023_3703_MOESM12_ESM.docx]

**Supplementary Table S12. Causal relationship between metabolites and bradycardia**

|  | **Exposure（Bacterial traits）** | **Methods** | **N.SNP** | ***P*.val** | **OR** | **95% CI-**  **lower** | **95% CI-**  **upper** |
| --- | --- | --- | --- | --- | --- | --- | --- |
| Diagnoses - secondary ICD10: R00.1 Bradycardia, unspecified \|\| id:ukb-b-11664 | N-acetylornithine | Inverse variance weighted | 9 | 0.0026 | 1.00 | 1.00 | 1.00 |
| Diagnoses - secondary ICD10: R00.1 Bradycardia, unspecified \|\| id:ukb-b-11664 | 2-stearoylglycerophosphocholine* | Inverse variance weighted | 7 | 0.0051 | 0.99 | 0.99 | 1.00 |
| Diagnoses - secondary ICD10: R00.1 Bradycardia, unspecified \|\| id:ukb-b-11664 | 1,6-anhydroglucose | Inverse variance weighted | 4 | 0.0062 | 1.00 | 0.99 | 1.00 |
| Diagnoses - secondary ICD10: R00.1 Bradycardia, unspecified \|\| id:ukb-b-11664 | Serotonin (5HT) | Inverse variance weighted | 7 | 0.0072 | 0.99 | 0.99 | 1.00 |
| Diagnoses - secondary ICD10: R00.1 Bradycardia, unspecified \|\| id:ukb-b-11664 | X-02973 | Inverse variance weighted | 4 | 0.0099 | 1.02 | 1.00 | 1.03 |
| Diagnoses - secondary ICD10: R00.1 Bradycardia, unspecified \|\| id:ukb-b-11664 | X-12729 | Inverse variance weighted | 3 | 0.0121 | 1.00 | 1.00 | 1.00 |
| Diagnoses - secondary ICD10: R00.1 Bradycardia, unspecified \|\| id:ukb-b-11664 | X-04498 | Inverse variance weighted | 7 | 0.0129 | 1.01 | 1.00 | 1.01 |
| Diagnoses - secondary ICD10: R00.1 Bradycardia, unspecified \|\| id:ukb-b-11664 | Tryptophan betaine | Inverse variance weighted | 4 | 0.0133 | 1.00 | 1.00 | 1.00 |
| Diagnoses - secondary ICD10: R00.1 Bradycardia, unspecified \|\| id:ukb-b-11664 | X-14374 | Inverse variance weighted | 5 | 0.0169 | 0.99 | 0.99 | 1.00 |
| Diagnoses - secondary ICD10: R00.1 Bradycardia, unspecified \|\| id:ukb-b-11664 | Taurolithocholate 3-sulfate | Inverse variance weighted | 4 | 0.0202 | 1.00 | 1.00 | 1.01 |
| Diagnoses - secondary ICD10: R00.1 Bradycardia, unspecified \|\| id:ukb-b-11664 | X-12749 | Inverse variance weighted | 21 | 0.0236 | 1.01 | 1.00 | 1.01 |
| Diagnoses - secondary ICD10: R00.1 Bradycardia, unspecified \|\| id:ukb-b-11664 | Free cholesterol in large LDL | Inverse variance weighted | 13 | 0.0244 | 1.00 | 1.00 | 1.00 |
| Diagnoses - secondary ICD10: R00.1 Bradycardia, unspecified \|\| id:ukb-b-11664 | X-03003 | Inverse variance weighted | 3 | 0.0253 | 1.01 | 1.00 | 1.02 |
| Diagnoses - secondary ICD10: R00.1 Bradycardia, unspecified \|\| id:ukb-b-11664 | X-14977--vanillin | Inverse variance weighted | 3 | 0.0270 | 0.99 | 0.99 | 1.00 |
| Diagnoses - secondary ICD10: R00.1 Bradycardia, unspecified \|\| id:ukb-b-11664 | Total cholesterol in large LDL | Inverse variance weighted | 12 | 0.0347 | 1.00 | 1.00 | 1.00 |
| Diagnoses - secondary ICD10: R00.1 Bradycardia, unspecified \|\| id:ukb-b-11664 | Total cholesterol in medium LDL | Inverse variance weighted | 11 | 0.0396 | 1.00 | 1.00 | 1.00 |
| Diagnoses - secondary ICD10: R00.1 Bradycardia, unspecified \|\| id:ukb-b-11664 | Indolepropionate | Inverse variance weighted | 10 | 0.0415 | 1.00 | 0.99 | 1.00 |
| Diagnoses - secondary ICD10: R00.1 Bradycardia, unspecified \|\| id:ukb-b-11664 | Erythronate* | Inverse variance weighted | 20 | 0.0438 | 1.01 | 1.00 | 1.01 |
| Diagnoses - secondary ICD10: R00.1 Bradycardia, unspecified \|\| id:ukb-b-11664 | 1,5-anhydroglucitol (1,5-AG) | Inverse variance weighted | 14 | 0.0439 | 1.00 | 0.99 | 1.00 |
| Diagnoses - secondary ICD10: R00.1 Bradycardia, unspecified \|\| id:ukb-b-11664 | 1-arachidonoylglycerophosphoinositol* | Inverse variance weighted | 9 | 0.0471 | 1.01 | 1.00 | 1.01 |
| Diagnoses - secondary ICD10: R00.1 Bradycardia, unspecified \|\| id:ukb-b-11664 | Glutamine | Inverse variance weighted | 6 | 0.0476 | 1.00 | 1.00 | 1.00 |
