# Supplementary material for: Putative causal relations among gut flora, serums metabolites and arrhythmia: a Mendelian randomization study
Source: BMC Cardiovasc Disord. 2024 Jan 11;24:38. doi: 10.1186/s12872-023-03703-z (PMC10782588; doi:10.1186/s12872-023-03703-z)
Supplement: Supplementary file 13 — Additional file 13: Supplementary Table S13. Causal relationship between metabolites and atrioventricular block. [file 12872_2023_3703_MOESM13_ESM.docx]

| **Outcome** | **Exposure（Bacterial traits）** | **Methods** | **N.SNP** | ***P*.val** | **OR** | **95% CI-**  **lower** | **95% CI-**  **upper** |
| --- | --- | --- | --- | --- | --- | --- | --- |
| AVBLOCK | X-12230 | Inverse variance weighted | 3 | 0.0338 | 0.41 | 0.18 | 0.93 |

**Supplementary Table S13. Causal relationship between metabolites and atrioventricular block**
